# Current and Developing Lymphatic Imaging Approaches for Elucidation of Functional Mechanisms and Disease Progression

**DOI:** 10.1007/s11307-023-01827-4

**Published:** 2023-05-17

**Authors:** Arjun Aron, Cristina Zavaleta

**Affiliations:** 1 Department of Biomedical Engineering, University of Southern California, 1042 Downey Way, Los Angeles, CA 90089 USA; 2 Michelson Center for Convergent Bioscience, University of Southern California, 1002 Childs Way, Los Angeles, CA 90089 USA

**Keywords:** Lymphatic system, Lymphatic imaging, Molecular imaging, Lymphoscintigraphy, Lymphedema, Sentinel lymph nodes

## Abstract

Study of the lymphatic system, compared to that of the other body systems, has been historically neglected. While scientists and clinicians have, in recent decades, gained a better appreciation of the functionality of the lymphatics as well as their role in associated diseases (and consequently investigated these topics further in their experimental work), there is still much left to be understood of the lymphatic system. In this review article, we discuss the role lymphatic imaging techniques have played in this recent series of advancements and how new imaging techniques can help bolster this wave of discovery. We specifically highlight the use of lymphatic imaging techniques in understanding the fundamental anatomy and physiology of the lymphatic system; investigating the development of lymphatic vasculature (using techniques such as intravital microscopy); diagnosing, staging, and treating lymphedema and cancer; and its role in other disease states.

## Introduction

The lymphatic system plays several varied, important roles in the function of the human body. On a daily basis, it takes up several liters of fluid that extravasates from the capillaries into the interstitial space and returns it to the circulation [1]. Lymphatic vasculature plays a key role in the immune system, transporting antigens and antigen-presenting cells to lymph nodes, resulting in detection and response to threats [1, 2]. Lacteals, a set of lymphatic vessels in the microvilli of the intestine, take up ingested lipids that cannot enter the bloodstream directly, providing a path for them to ultimately enter circulation [3, 4]. Due to their vital role as a trafficking system, the lymphatics can cause or exacerbate several debilitating diseases when a physiological irregularity arises in the body. For instance, lymphedema, a painful condition resulting in swelling of the arms and legs, is directly caused by an inability of the lymphatic vasculature to adequately drain interstitial fluid [5]. Lymphatic vasculature, in addition to the cardiovascular system, also provides routes for cancer to metastasize, a critical event resulting in much higher mortality rates for patients [1, 6].

**Fig. 1:**
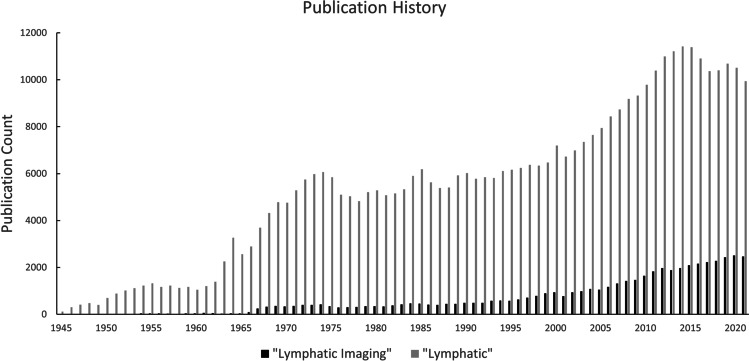
Academic Interest in lymphatics and lymphatic imaging from 1945 to 2021 in the PubMed Database. A) New entries for “lymphatic” (plotted in grey) and “lymphatic imaging” (plotted in black)

Despite the significance of the lymphatic system in both day-to-day function and disease progression, knowledge regarding its inner workings has historically lagged behind that of its sanguine counterpart. This disconnect is perhaps most striking when considering soon-to-be clinicians’ level of exposure to the topic during their training; a 2004 article by Rockson *et al*. reported that the average US medical student received less than 30 min of instruction regarding the lymphatic system during the course of their studies [7]. In recent decades, a surge of academic interest in the lymphatics has helped fill this knowledge gap. Interest in lymphatic imaging has helped lead the way, with over 27,000 new entries into the PubMed database since the turn of the millennium (Fig. 1). Despite this recent interest, lymphatic imaging remains as a relatively small proportion of all conducted lymphatic research. Additional work on the imaging front needs to be done to better understand the lymphatic system and its role in various disease states. In this review, we discuss several current and developing lymphatic imaging approaches for assessment of lymphatic function and development, as well as imaging methods used for diagnosing, staging, and planning the treatment of associated diseases like cancer and lymphedema. We also discuss the current limitations of some of these approaches and areas of improvement for future techniques to ultimately improve patient outcomes.

### Imaging for Lymphatic Architecture and Functionality

The most common method for observing the lymphatics, particularly drainage patterns, is lymphoscintigraphy. In lymphoscintigraphy, a radiotracer (most commonly ^99m^Tc-sulfur colloid) is injected intradermally into the affected regions, typically the arms or the legs. The colloid emits gamma rays which can be detected using a gamma camera, which uses the detected radiation to produce an image [8]. Several other techniques similar to lymphoscintigraphy also exist, including near-infrared fluorescence (NIRF) lymphangiography and MRI lymphangiography. Additional information about some of these techniques is included in the “Imaging Techniques for Lymphedema Diagnosis and Treatment” section.

Visualization of the truncal lymphatics specifically can also leverage one of the intrinsic functions of the lymphatic system. The lymphatics play a key role in transporting re-packaged dietary lipids in the form of chylomicrons from the small intestine to the heart, where they enter systemic circulation [9]. The use of radioactive lipid analogs have been administered via oral gavage in animal models to better observe mesenteric and truncal lymphatics for varied applications, such as studying ovarian cancer [4].

The use of modern imaging techniques, such as intravital microscopy (particularly using multiphoton microscopy), have recently helped shed additional light on the functionality of the lymphatic system. Multiphoton microscopy involves using a pulsed laser to shoot two or more lower-energy photons near-simultaneously at a fluorophore, exciting it to a higher-energy state. The released photon following the transition of the fluorophore to its ground state is detected and used to form the microscopy images. This technique allows for high-resolution real-time imaging at greater depths, making it ideal for intravital imaging applications [10].

The use of multiphoton imaging has been especially beneficial for observing the role of the lymphatics in the immune response. Several studies have demonstrated important lymphatic phenomena occurring *in vivo*, such as the intralymphatic “crawling” mechanism mediating the movement of T cells from inflammation sites to draining nodes and macrophage presentation of antigens to B cells [11, 12]. Steven *et al*. used suture placement in BALB/c mice corneas to induce neovascularization and inflammation and subsequently observed migration of immune cells such as T cells and macrophages into lymphatic vasculature [13]. Another intravital study using a similar corneal implant suture methodology on transgenic mice expressing green fluorescent protein (GFP) under the *Prox-1* promoter sought to observe more fundamental lymphatic processes. In this study, researchers were able to observe lymphatic angiogenesis and valvulogenesis, which they noted originated in existing limbal vessels. Additionally, they were able to observe that lymphatic elongation was a result of stalk cell migration, a phenomenon that is impossible to detect using traditional methods. These observations all offer important glimpses into lymphatic propagation, particularly in inflammatory/diseased states which could ultimately prove useful in therapeutic approaches with further study [14].

**Fig. 2:**
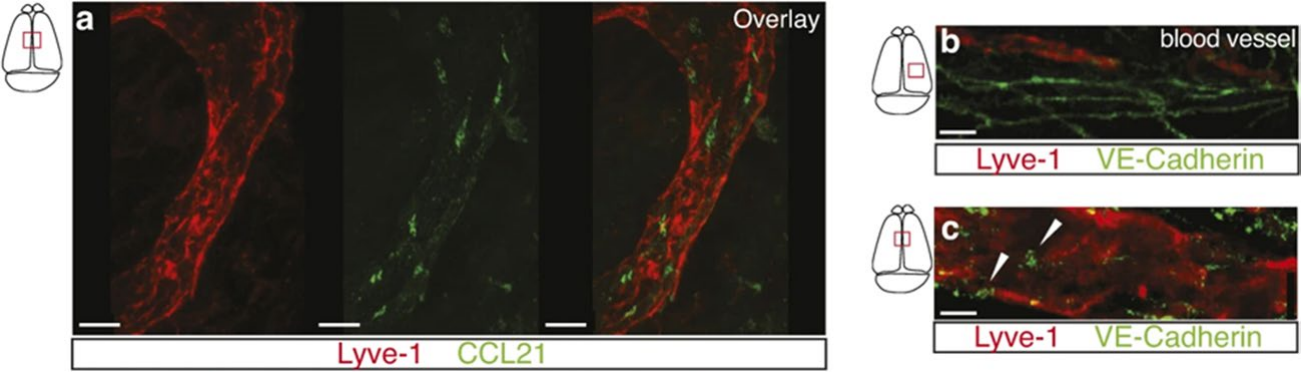
Initial lymphatic features of meningeal lymphatic vessels. **a** Representative images of CCL21 and Lyve-1 labeling of the meningeal lymphatic vessels (scale bars, 10 μm). **b**, **c** Representative images of VE-Cadherin and Lyve-1 staining on meningeal blood vessels (**b**) and meningeal lymphatic vessels (**c**), arrowheads point to the VE-Cadherin aggregates; scale bars, 10 μm. (Adapted with permission from Louveau et al., 2015, ©2015, Nature published by Springer Nature)

These intravital techniques have also lent themselves to enhancing our foundational understanding of lymphatic anatomy. Previously, it had long been thought that the central nervous system lacked lymphatic vessels. However, following drainage comparison studies using Qdot655 (a fluorescent nanoparticle quantum dot) in the meninges of mice, Louveau *et al*. were able to conclude that non-cardiac vessels lining the dural sinuses did in fact drain the cerebrospinal fluid (Fig. 2). These vessels were subsequently affirmed to be lymphatic channels based on histologic analysis and identification of various lymphatic biomarkers. Immunohistochemical analysis also revealed that these vessels could carry leukocytes such as T lymphocytes and MHCII+ cells [15].

**Fig. 3:**
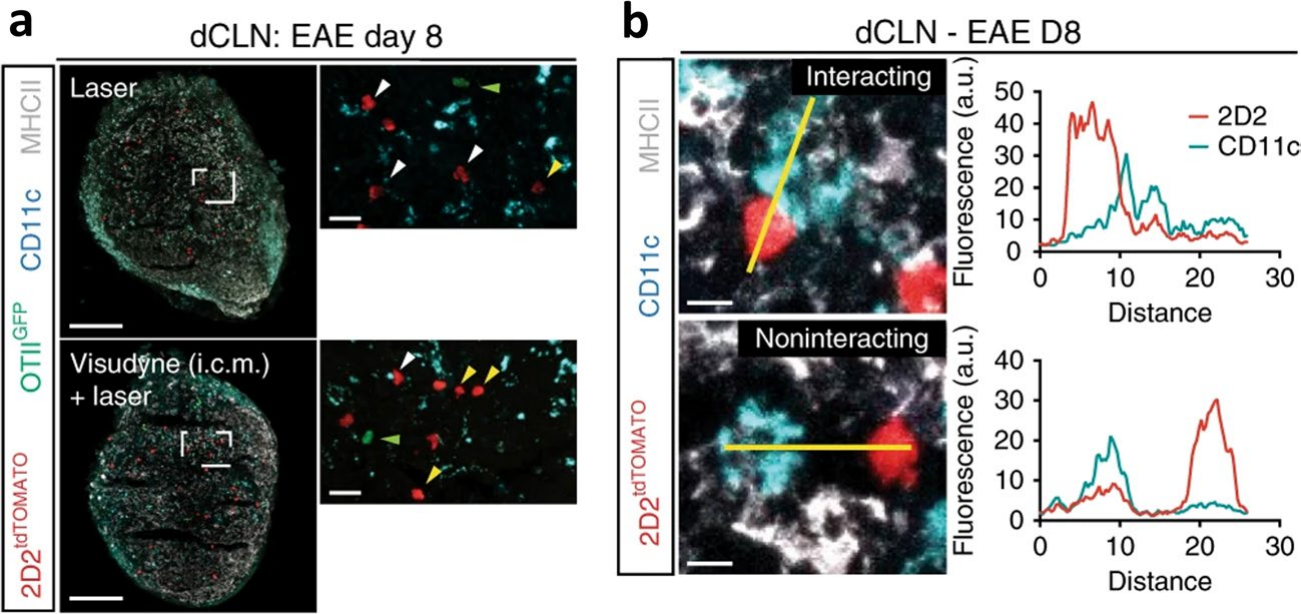
**a** Representative images of MOG-specific T cells (2D2) and OVA-specific T cells (OTII) in the dCLNs of mice treated with laser and Visudyne (i.c.m.) + laser at day 8 after EAE induction. Green arrowheads, OTII T cells. Yellow arrowheads, 2D2 cells not in contact with CD11c^+^ cells; white arrowheads, 2D2 cells in contact with CD11c^+^ cells. Scale bar, 150 μm; insets, 25 μm. Representative of 5 independent mice per group. **b** Representative images and associated profile plots of MOG-specific T cells (2D2, red) in close contact (top) or not in contact (bottom) with CD11c^+^ cells (cyan) in the dCLNs of mice treated with laser and Visudyne (i.c.m.) + laser 8 d after EAE induction. Scale bar, 10 μm. Representative of 5 independent mice per group. (Adapted with permission from Louveau et al., 2018, ©2018, Nature Neuroscience published by Springer Nature)

A subsequent 2018 study by Louveau *et al.* further investigated these newly discovered lymphatic vessels. Using transgenic mice models, authors were able to demonstrate that the lymphatic vasculature played a key role in T cell drainage. In the same study, the authors also conducted an investigation using experimental autoimmune encephalomyelitis (EAE) in mice as a model for multiple sclerosis (MS) [16]. When they surgically ablated the lymphatic vessels, the authors found a reduction in the EAE pathology (Fig. 3). These results indicated that these meningeal lymphatics played an important role in the activation of encephalitogenic T cells, thereby playing a key role in regulating neuroinflammatory responses and subsequently serving as a target for future therapeutic interventions [17]. The significance of works investigating the meningeal lymphatics in mice models was further amplified when Absinta *et al.* were able to image meningeal lymphatic vessels in marmoset monkeys as well as humans using MRI with gadolinium-based contrast agents. More specifically, they were able to determine that the topographies of these visualized vessels matched those described in the prior mouse studies [18]. This breakthrough observation via noninvasive methods opens the door to further study of CNS lymphatics and investigation of how they might be involved in the manifestation of various neurological disorders.

### Imaging Techniques for Lymphedema Diagnosis and Treatment

Lymphedema is a chronic, debilitating disease estimated to affect approximately 200 million people around the world [19]. It can be categorized into two main types. Primary lymphedema is genetic and quite rare. Onset typically occurs during childhood or adolescence and approximately 1 in 100,000 are affected by the condition [20]. Secondary lymphedema, in contrast, is much more common and is estimated to effect 2 to 3 million patients in the USA alone [21, 22]. Also, unlike primary lymphedema, it is an acquired condition, with onset tending to occur in adulthood [5, 23]. It is the direct result of significant damage to lymphatic structures, resulting in their inability to maintain fluid homeostasis in tissues. In developing nations, the primary causes of secondary lymphedema are infections resulting in inflammation and fibrosis of lymphatic channels and nodes. This is not the case in developed nations, where the requisite lymphatic trauma is often the result of medical intervention, particularly surgical and radiation-based treatments of cancer [5, 21].

In addition to these primary types, lymphedema can be further sub-categorized into different stages. The International Society of Lymphology categorizes these stages as follows. In Stage 0 lymphedema, there are no signs readily apparent in a clinical examination, although there is still abnormal lymphatic flow and drainage. Stage 1 is characterized by mild swelling which can be mitigated by limb elevation; pitting may also be present at this stage. Stage 2 lymphedema can no longer be alleviated by limb elevation. Early phases of Stage 2 are also characterized by pitting edema, while late phases of Stage 2 may or may not involve pitting but do result in fibrosis and irreversible damage to the tissue. Stage 3 consists of lymphostatic elephantiasis, the most severe form of lymphedema. At this point, swelling is extreme, resulting in large limbs and trophic skin changes, such as leathery, and potentially warty, skin [5, 23–25].

Although, in many instances, lymphedema can be diagnosed through physical examination, its manifestation cannot be confirmed without the use of a more specific diagnostic test, typically one that is imaging-based [23]. This confirmation can be especially important in improving patient outcomes because lymphedema is often misdiagnosed in initial screenings. In fact, a 2011 study by Schook *et al*. found that approximately one-fourth of all pediatric patients referred to their clinic with “lymphedema” were suffering from a different condition [26]. A 2015 study by Maclellan *et al.* noted a similar prevalence of initial misdiagnosis in a pediatric and adult patient population [27]. The use of diagnostic tests can also help illustrate the pathophysiology of each patient’s lymphedema, helping stage the disease and affecting treatment choice.

The current “gold-standard” for diagnosis confirmation is lymphoscintigraphy. If the lymphatics are functioning normally, then the colloid will be taken up by lymphatic vessels and drain to downstream nodes. Lymphoscintigraphy can be used qualitatively or quantitatively to assess lymphatic function. Qualitative approaches rely on making a diagnosis based on visual assessment of lymphatic vasculature and nodes, and their ability to uptake the radiotracer. In contrast, quantitative approaches utilize the use of more defined metrics such as time of uptake and clearance of the radiotracer from the limb of interest or from the injection site [24, 28].

**Fig. 4:**
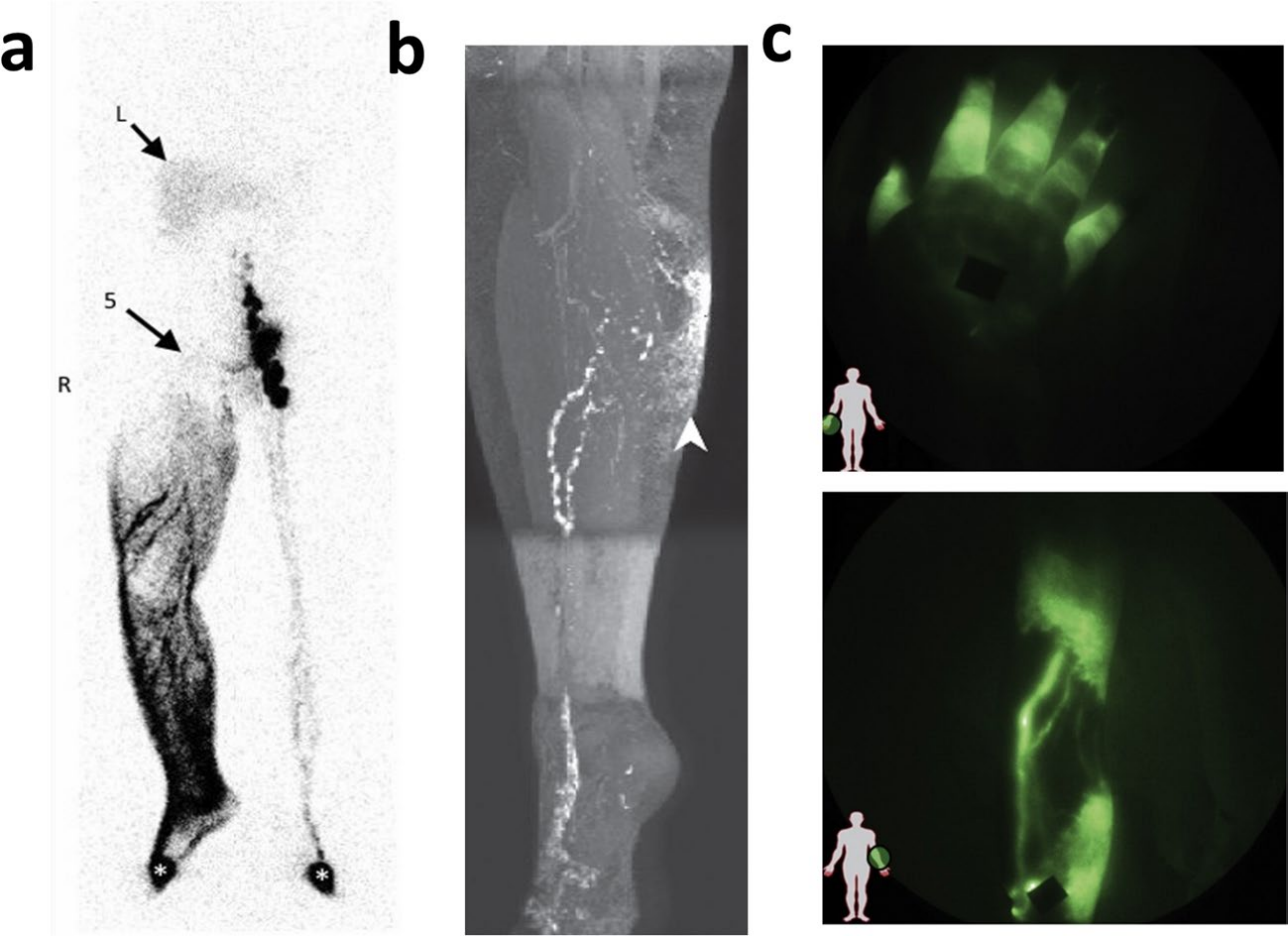
**a** Traditional lymphoscintigraphy shows the consequences of extensive inguinoiliac lymphadenodysplasia (the inguino-iliac lymph nodes are not visible, arrow 5 on the right side with the tracer flowing into lymphatic vessels and the superficial lymphatic collateralization network up to the root of the limb. Arrows with L indicate the liver, wherein radiocolloids are taken up when they have reached the systemic circulation. (Adapted with permission from Roman et al., 2018, ©2018, World Journal of Surgical Oncology published by Springer Nature) **b** T1-weighted gradient echo MRI maximum intensity projection image of a 55-year-old woman with a history of ovarian cancer and pelvic lymphadenectomy who presented with right leg edema. After intracutaneous administration of a contrast agent into the web spaces of the right foot MRI imaging reveals a large lymphatic channel ascending the anterior calf and dermal backflow to the medial upper calf (arrowhead). Dermal backflow is intermittently identified with MRI in some patients with secondary lymphedema. Note the increase in spatial resolution as compared with lymphoscintigraphy. (Adapted with permission from Lee et al., 2022, ©2022, RadioGraphics published by RSNA) **c** Near infrared fluorescence images of diseased lymphatics (top) showing retrograde flow in symptomatic hand, and (bottom) tortuous vessels in symptomatic leg. Black spots are covered injection sites. (Adapted with permission from Rasmussen et al., 2009, ©2009, Current Opinion Biotechnology published by Elsevier)

Lymphoscintigraphy’s status as the go-to imaging approach for lymphedema diagnosis has been well-earned due to its consistent accuracy in detecting lymphedema for decades. A 1988 study using lymphoscintigraphy to assess lymphatic function in a group of 238 patients demonstrated that qualitative analysis of the image alone allowed researchers to identify flow abnormalities in 70.1% of studied extremities and subsequent quantitative analysis allowed for identification in all extremities [29]. Similarly, a 1989 study conducted by Gloviczki *et al*., in which the authors performed lymphoscintigraphy on 190 patients, found that semiquantitative evaluation of the lymphatics allowed them to detect lymphedema with a sensitivity of 92% and a specificity of 100% [30]. A more recent 2017 study with 227 patients found that lymphoscintigraphy had a 96% sensitivity and 100% specificity, reaffirming and marking a slight improvement on the historical trend [31]. In 2018, a study used traditional lymphoscintigraphy to differentiate primary versus secondary lower extremity lymphedema after surgical lymphadenectomy. The retrospective analysis revealed that the appearance of lower limb lymphedema was not related to cancer therapeutic intervention but points to a primary lymphatic disease prior to treatment (Fig. 4a) [32].

Despite this great accuracy in detecting true negatives and positives, lymphoscintigraphy does have some drawbacks. First and foremost, lymphoscintigraphy has poor spatial resolution [33]. This may not have a significant impact on its ability to detect the presence of lymphedema, but it does mean that lymphoscintigraphy cannot provide much anatomical information, placing limitations on how much information it can provide clinicians about the specific disease states of individual cases. Lymphoscintigraphy also lacks standardization, particularly regarding quantitative analysis of results. There are no “standardized” sets of drainage times or even acquisition time points by which clinicians can conclusively diagnose lymphedema [34]. Instead, they largely rely on conducting bilateral lymphoscintigraphy, and compare drainage times and patterns between the suspected diseased and healthy limbs. This introduces another variable into the diagnosis process and can open the door to inconsistency in diagnosis from center to center and clinician to clinician. Lastly, although quantitative lymphoscintigraphy does allow for increased sensitivity relative to qualitative methods, lymphoscintigraphy images can still be “normal” for patients in very early stages of the disease process [35]. This means that lymphoscintigraphy may be unable to detect lymphedema early enough, resulting in detection only when more extensive physiological abnormalities such as dermal backflow are manifested more thoroughly.

The use of techniques such as MR lymphangiography instead of lymphoscintigraphy represents a paradigm shift in philosophy for lymphedema diagnosis. With its poor resolution, lymphoscintigraphy provides diagnosis primarily based on lymphatic functionality. MR lymphangiography, on the other hand, uses direct visualization of the lymphatic vasculature to determine whether there are issues in lymphatic drainage that could be attributed to lymphedema. Greater anatomical resolution makes MR lymphangiography useful for gaining a better understanding of pathophysiology of disease (Fig. 4b) [36, 37]. This improved resolution, also has, in several recent studies, been used in planning clinical procedures involving lymphatic vasculature such as lymphaticovenous anastomosis, a surgical technique used in the treatment of lymphedema [38, 39]. For the purposes of lymphedema diagnosis, MR lymphangiography is typically performed using a 1.5 T magnet (although, more recently, 3.0 T magnets have also been used) and injection of gadolinium-based contrast agents into the interdigital webbings of the hand and the foot [36, 40]. More recently, various studies are also beginning to apply non-contrast approaches. The subsequent images allow for visualization of lymphatic vessels, more specifically delayed lymphatic drainage, lymphatic dilation as a result of potential obstruction, and tortuous vessels [36].

Given this high degree of anatomic detail, the question then becomes whether this additional information proves useful for MR lymphangiography to match or surpass lymphoscintigraphy in lymphedema detection sensitivity and specificity. To this point, the answer appears to be no. While some recent studies have indicated that MR lymphangiography’s sensitivity could match that of lymphoscintigraphy, this is often not the case. In general, lymphoscintigraphy appears to consistently have higher sensitivity and specificity than MR lymphangiography. A representative, comparative study conducted by Weiss *et al*., published in 2014, found that while the modalities did have some association (correlation coefficient = 0.62), MR lymphangiography only achieved a sensitivity of 68% and a specificity of 91% [40]. Compared to several large lymphoscintigraphy studies conducted over the past several decades that achieved sensitivities above 90% and specificities close to 100%, the diagnostic performance of MR is lacking [30, 31]. Despite this, MR lymphangiography still remains an extremely effective tool for visualizing lymphatic anatomy, and, in the future, could prove to be a fantastic supplementary tool for planning lymphatic-based procedures.

In keeping with the same paradigm of anatomy-based diagnosis of lymphedema, ultrasound techniques also present a potential alternative to lymphoscintigraphy. Like MR lymphangiography, ultrasound relies primarily on imaging soft tissue to make lymphedema diagnoses. Unlike MR techniques (and lymphoscintigraphy, for that matter) ultrasound is quite cheap, quick, and readily available. It also does not require the injection of any contrast agents. This accessibility, combined with the relative simplicity of the actual image acquisition process, has made ultrasonography a mode of interest for lymphedema diagnosis [41]. Ultrasound is particularly useful for identifying volumetric changes, and so clinicians typically look for thickening of the dermis, subcutaneous layer, and muscle. The specific changes in these different layers varies between cases of primary and secondary lymphedema, indicating that ultrasound can serve as an effective tool for further characterization of patient disease states [42]. To this point, the use of duplex ultrasound techniques have been shown to assist in staging lymphedema. A 2013 study by Suehiro *et al*. showed that lymphedema could be reliably staged by measuring skin thickness, subcutaneous tissue thickness, and subcutaneous tissue echogenicity. However, the authors of the study noted difficulty in measuring subcutaneous tissue and skin thickness for later stage patients, and concluded that tissue echogenicity should be the primary ultrasound measurement used for diagnostic purposes [43]. Previous studies have also compared the efficacy of lymphoscintigraphy with that of ultrasound for lymphedema diagnosis. A 2021 paper with 14 enrolled lymphaticovenous anastomosis patients found that ultrasound had a 94.6% diagnostic accuracy and actually correctly diagnosed lymphedema in 39 of 54 leg areas where lymphoscintigraphy failed to detect it [44]. A 2010 study utilized both lymphoscintigraphy and duplex ultrasound to effectively distinguish between leg lymphedema and other edema abnormalities [45]. At the very least, the current literature warrants further exploration of using ultrasound and lymphoscintigraphy in concert to diagnose and characterize lymphedema.

One of the most recent lymphedema diagnosis methods is the use of near-infrared fluorescence (NIRF) imaging. As the name suggests, NIRF imaging utilizes fluorophores (the most common one being indocyanine green, or ICG) that are readily excited in the near infrared range to generate fluorescent signals that are then detected and used to construct images of the tissue containing the imaging agents [46]. While this technique itself, and particularly its clinical application to lymphedema diagnosis, is relatively modern, ICG has a long history of use in clinical diagnostics, dating back to 1956 [47]. NIRF imaging, although depth-limited at about 2–4 cm, provides improved spatial and temporal resolution relative to many common lymphatic imaging techniques including lymphoscintigraphy and MR lymphangiography [4, 46]. This allows for enhanced visualization of the lymphatic vessel anatomy. In fact, NIRF can allow for non-invasive observation of lymphatic phenomena such as pumping in both pre-clinical and clinical settings, making it particularly useful for readily identifying issues with lymphatic transport [48]. In regards to lymphedema, signs of lymphatic irregularities such as dermal backflow and tortuous vessels can then be used to make a diagnosis of lymphedema in the limbs (Fig. 4c) [24]. Additionally, NIRF imaging is quicker to perform and cheaper to execute in comparison to other techniques such as MR and PET, making it a particularly appealing option in clinical settings. While some studies have indicated that NIRF imaging could be comparable or even represent an improvement in some instances to lymphoscintigraphy in lymphedema diagnosis, given the decades-long track history of lymphoscintigraphy as a very effective tool for this purpose, additional work is required to draw any definitive conclusions about the comparability of these techniques [35, 49].

Several of these (and other) lymphatic imaging techniques have also been utilized for planning surgical treatments of lymphedema, such as lymphaticovenous anastomosis, or LVA. LVA is a microsurgical technique that involves anastomosing lymphatic vessels with surrounding venous structures; this allows for the lymphatic fluid to re-enter the circulating vasculature, and thereby prevents its build-up in the interstitial space [50]. Historically, lymphoscintigraphy has been one of the most common imaging modalities used for preoperative determination of incision locations as well as assessment of the efficacy of the procedure in reducing the symptoms of lymphedema. Intraoperatively, various blue fluorescent dyes have been used to help identify vessels for use in anastomosis [51].

**Fig. 5:**
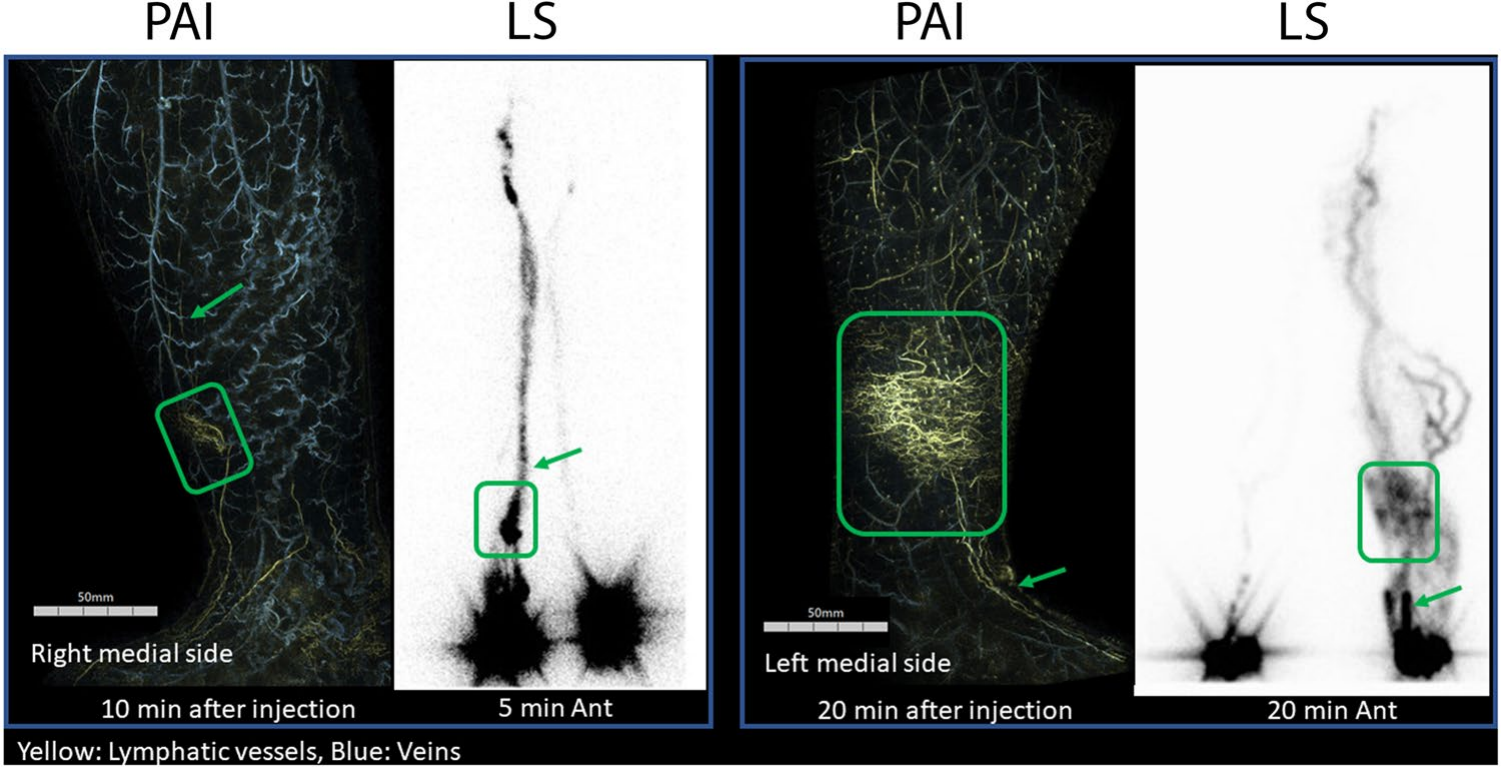
Comparative images between traditional lymphoscintigraphy (LS) and photoacoustic imaging (PAI). Dermal backflow (DBF) was visualized similarly using both techniques. Both show representative cases of type 3 lymphedema with dermal back flow in the early phase of lymphoscintigraphy. Each DBF is surrounded by a *square*, and the linear lymphatic vessels proximal and distal to it (*arrows*) can be seen in both examinations. (Adapted with permission from Watanabe et al., 2022, ©2022, Journal of Vascular Surgery: Venous Lymphatic Disorders, published by Elsevier)

Photoacoustic (optoacoustic) imaging is a relatively newer imaging technique where light is used to photothermally heat a highly absorbing material which, as a result, expands locally to produce an acoustic pressure wave that can be measured using an ultrasound transducer [52]. It can be used in combination with a highly absorbing dye or nanoparticle to create a contrast-enhanced image. It has recently been used as a possible alternative for lymphedema staging [53]. Lymphatic vessels were visualized using a highly absorbing contrast agent (ICG) that was injected into the interdigital space of the foot. The lower extremities were examined using photoacoustic imaging (Fig. 5) for staging lymphedema and compared with those obtained using traditional lymphoscintigraphy. The number of lymphatic vessels visualized using photoacoustic imaging was similar to that of lymphoscintigraphy. The investigators concluded that the findings from photoacoustic imaging could be useful for lymphedema staging as they corresponded well with the clinical gold standard lymphoscintigraphy images [53].

Recently, other techniques, particularly the use of NIRF dyes such as ICG have become a subject of interest in the lymphedema imaging. ICG has been used for pre-operative determination of incision, assessment of lymphedema symptoms both pre-operatively and post-operatively, and even intraoperative guidance, replacing the need for another dye to provide contrast [54, 55]. However, as mentioned earlier, ICG, like all NIRF agents, is depth-limited. As a result, in cases where there is abundant sub-cutaneous tissue (like in obese patients), visualization of lymphatics can be difficult via ICG, particularly for assessment of the efficacy of the treatment. In these cases, it may be more useful to pair imaging modalities, like using lymphoscintigraphy in conjunction with ICG imaging [56]. The use of these new imaging modalities for these treatment applications represents potential area for growth in the field, with more and more recent work focused on the topic. For instance, a recent study by Seki *et al*. investigated the use of real-time video lymphography with ICG to help guide LVA and achieve better surgical outcomes. While work like this is quite new and needs to be further investigated to assess its efficacy compared to previous methods, this represents a new and exciting direction for the field of lymphatic imaging within the context of lymphedema assessment and treatment [55].

### Cancer and Sentinel Lymph Node Mapping

The significance of the lymphatics in the progression of cancer cannot be overstated. Lymphatic vessels often serve as a pathway for cancer cells to migrate from the primary tumor location and metastasize, and so imaging lymphatic structures can help serve as a crucial step in staging cancer and planning its treatment [6]. While the lymphatics have been primarily viewed as passive contributors to the progression of the disease state, recent studies utilizing lymphatic biomarkers and *in vivo* imaging techniques have indicated that lymphatic activity, specifically tumor-mediated lymphangiogenesis, could play a more active role in the process of metastasis [57]. As a result, current and future imaging techniques will serve a crucial role in staging cancer, determining effective treatment plans, and providing a deeper fundamental understanding of cancer as a disease state.

One of the primary areas of interest for cancer investigation is the identification of lymph node metastases. Sentinel lymph nodes (SLNs), the primary draining nodes for a particular region in the body, typically represent the first locations where cancer metastasis from a given region occur, thereby serving as a key indicator for disease staging and prognosis. Specifically identifying sentinel lymph nodes also helps narrow down potential locations of metastasis, potentially eliminating the need for broadscale clearance of surrounding lymphatic structures. This knowledge can allow for the removal of just a few selective nodes during surgery, rather than broad-scale excision of all draining lymphatic channels in a nodal basin [58]. Modern sentinel lymph node dissection (SLND) techniques are not only less disruptive to lymphatic drainage, but also demonstrate comparable clinical effectiveness in comparison to more indiscriminatory nodal removal techniques. For instance, a clinical trial conducted by Giuliano *et al*. in 2011 found that there was no significant difference in 5-year overall survival and 5-year disease-free survival between SLND and ALND (axillary lymph node dissection) treatment groups of invasive breast cancer patients [59]. The specificity afforded by this technique can greatly reduce disruption of the lymphatics, helping alleviate iatrogenic lymphedema and other potential morbidities [60, 61]. Among the first documented instances of sentinel lymph node biopsy were described by Gould *et al.* in cases of parotid cancer in 1960 [62]. While the authors of this initial study remarked that identifying and accessing the sentinel lymph node was quite straightforward for the parotid, this is not necessarily the case for tumors of other glands and organs. This resulted in significant interest in the use of various imaging contrast agents for this purpose.

Among the most common imaging tools used for the purposes of lymphatic mapping for subsequent SLND are lymphoscintigraphy and visible contrast provided by blue dyes, particularly isosulfan blue, patent blue, and methylene blue. Lymphoscintigraphy was first utilized for the purposes of identifying sentinel lymph nodes in 1977 by Dr. Ramon Cabañas, who used it in patients with penile carcinoma. The Cabañas study was able to demonstrate that other nodes in the region, including the iliac and inguinal-femoral nodes did not participate in drainage prior to participation of the sentinel lymph node, reaffirming the significance of the sentinel lymph node as an indicator of metastasis through lymphatic channels [63]. Morten *et al.*, in a 1992 study, utilized lymphoscintigraphy to preliminarily identify draining nodes in breast cancer patients; the authors also used isosulfan blue and patent blue-V dyes to help map the lymphatics intraoperatively. Researchers were able to use lymphoscintigraphy to readily identify the sentinel lymph node in 194 of the 237 subjects (81.9%) and subsequently analyze these nodes to determine whether metastasis had occurred. Like in the Cabañas study, this allowed the authors to determine whether there would be any benefit to performing additional lymphadenectomies, allowing them to avoid major lymphatic disruption and reducing the likelihood of the patient suffering subsequent edema [64].

In 1994, Giuliano *et al*. were the first to demonstrate the efficacy of blue dye injections (specifically isosulfan blue) for sentinel lymph node mapping. While the authors were only able to identify the sentinel lymph nodes in 114 of 174 cases (65.5%), a significant portion of this difficulty could be attributed to the inexperience of the surgeons at performing this novel (at least for the time) procedure. The learning curve of the surgeons was quite steep; however, for the first half of cases, the detection rate was a paltry 58.6%, but for the second half of cases, the detection rate increased to 72.4%. Using histopathology on sentinel lymph node and non-sentinel axillary lymph nodes specimens, the authors found that there was agreement of histology results in 95.6% of cases where surgeons were able to identify the sentinel lymph node. The overall sensitivity of the method was 88% and 100% for the study [65]. This study was incredibly consequential, as it provided credibility to blue dye mapping as an SLND technique and provided the impetus for further investigation of isosulfan blue and other visible dyes, such as methylene blue. A recent meta-analysis concerning the use of methylene blue for sentinel lymph node biopsy found that these procedures had an average identification rate of 91%, exceeding the minimum acceptable threshold of 85% as set by the American Society of Breast Surgeons to avoid complete ALND. However, the same meta-analysis also found that there was a high false negative rate [66, 67]. As a result, the current standard is to use lymphoscintigraphy in conjunction with a blue dye for SLND [68].

Since the general acceptance of lymphoscintigraphy and visible blue dye SLND techniques, there has been an increased interest in investigating other potential methods for performing sentinel lymph node biopsy; this investigation has also been informed in part by the fact that the radionuclides used in lymphoscintigraphy are difficult and expensive to acquire in comparison to simple dyes [69]. One common technique of focus has been the use of alternate dyes, particularly ICG. The efficacy of ICG has been, and continues to be, studied for use in sentinel lymph node biopsies for a range of cancers, including, but certainly not limited to, oral, gastric, lung, and breast cancer [70–73]. In an early study by Motomura *et al*., the authors found that they were able to successfully identify sentinel lymph nodes using ICG in 127 of 172 (73.8%) of cases, and of the 127 identified patients, there was agreement in the status between the sentinel and axillary nodes in 122 cases (96.1%) [74]. These results are quite comparable with the 1994 Giuliano *et al*. study, which was the first one to solely use blue dye injection (rather than blue dye in conjunction with radiotracer imaging) for the purposes of SLNB [65].

The results of more recent studies have helped bolster the case for ICG as a viable method for performing SLNB. Specifically, some studies have also highlighted the enhanced diagnostic efficacy of ICG over other optical imaging techniques. For instance, a 2017 retrospective study focusing on SLNB methods for breast cancer patients found that there was a statistically significant (*P* = 0.006) difference in fluorescence labeling of SLN’s, with general fluorescence due to ICG successfully identifying 100% of nodes, and methylene blue labeling identifying 88.3% [75]. Further investigating this comparison, another 2017 study found that ICG and radiotracer usage in fact had higher rates of SLN localization (98.0% and 97.8% respectively) in comparison to blue dyes (79.4%) [76]. In a 2018 clinical trial comparing the use of ICG and lymphoscintigraphy using ^99m^Tc, ICG was identified in 215 of 220 dissected nodes and radiotracer was identified in 172 of the nodes. Additionally, for all 99 patients included in the study, the use of the radiotracer in conjunction with ICG resulted in a 100% SLN detection rate, further suggesting the validity of ICG’s use as a contrast agent for SLNB [77].

As mentioned in the “Imaging Techniques for Lymphedema Diagnosis and Treatment” section, despite their high resolution, ICG and NIRF imaging techniques do have a significant drawback in that they are quite depth-limited and so best suited for superficial visualization. This has led to investigation in recent years of short-wave infrared (SWIR) or second near-infrared (NIR-II) fluorophores. SWIR/NIR-II, due to the longer wavelength 1000–1700 nm imaging window, results in reduced photon scattering, allowing for improved resolution at greater depths. Interestingly enough, early studies demonstrated that ICG had strong SWIR-range emission and could be a viable agent for this imaging strategy. In a 2017 study, Starosolski *et al.* demonstrated that NIR-II use of ICG could be used to effectively and non-invasively visualize sub-surface blood vessels in the hind limbs of mice. Furthermore, they demonstrated that the ICG-NIR-II usage had a contrast-to-noise ratio 5–8 times greater than that of IR-E1050 (a commercially available NIR-II agent) and ~ 1.5–2 times greater than that of ICG-NIR for the *in vivo* limb vessel imaging [78]. A 2018 study by Carr *et al*. was similarly able to showcase the capabilities of NIR-II, demonstrating extensive real-time visualization of several sub-surface processes in a mouse model, including hepatobiliary clearance, heart beats via intravital angiography, and clearance of ICG through dorsal lymphatic vessels and nodes. These promising results, in conjunction with ICG’s status as the only NIR dye approved for clinical use by the FDA, indicates strong potential for the use of SWIR/NIR-II with ICG for evaluation of drainage dysfunction and mapping of sentinel lymph nodes [79].

Studies involving SWIR/NIR-II have also investigated their use for cancer targeting and guiding surgical interventions. In the same study by Carr *et al*., the researchers also conjugated IRDye 800CW with trastuzumab, a tumor-targeting antibody. In a mouse model with implanted human breast cancer cells in the brain, the researchers were able to use SWIR imaging following injection of the dye-antibody conjugate to visualize the tumor non-invasively among the surrounding brain vasculature [79]. Similarly, a 2021 study by Swamy *et al*. was able to demonstrate that pi-conjugation extended ICG could be used in SWIR to visualize mouse vasculature and lymphatic structures non-invasively, and when the dye was conjugated with monoclonal antibodies, researchers were able to localize HER-2 and EGFR expressing breast tumors implanted in the mice [80]. Lastly, work by Cosco *et al*. was able to demonstrate the use of ICG along with two different flavylium heptamethine dyes to generate real-time three-color images of a mouse model non-invasively. The multiplexed nature of this approach presents exciting surgical possibilities; the various dyes could, for instance, be used to monitor draining lymphatics and circulatory vasculature simultaneously in a procedure to remove a tumor or sentinel lymph nodes [81]. While the use of SWIR/NIR-II is still relatively new, it represents an area ripe for further exploration and an opportunity to build on the pre-clinical and intraoperative imaging advantages of traditional NIR imaging.

Studies using traditional methods and even novel techniques have indicated using multiple imaging contrast agents tends to result in better identification of sentinel lymph nodes and improved mapping in general. For some studies, this has involved using a combination of two dyes, such as methylene blue and ICG, but most studies demonstrated improved results when using a combination of a dye (most popularly ICG) and a radiotracer [82]. This approach allows for a balancing of the deficiencies of both methods. ICG, while offering high resolution as a contrast agent for NIRF imaging, is quite leaky due to its low molecular weight, resulting in poor retention of it over time in lymph nodes in many clinical settings; this makes it ill-suited for pre-operative mapping. As such, using a radiocolloid with the NIRF agent can allow for effective localization pre-operatively while the ICG is able to provide clear SLN visualization through NIRF intraoperatively. The development of a hybrid tracer, ICG-[^99m^Tc]Tc-albumin nanocolloid, has resulted in improved SLN detection results relative to the usage of either ICG or the radiocolloid alone in several urologic and gynecologic applications, including a 2021 study by Sánchez-Izquierdo *et al*., investigating its uses for endometrial cancer [83, 84]. As the early returns on these hybrid methods suggest, development and clinical translation of imaging contrast agents that can allow for multimodal imaging applications would not only help provide more comprehensive information to physicians but would also simplify the entire process of lymphatic drainage assessment.

Nanoparticles are among the most promising candidates for these multimodal molecular imaging platforms. These particles can be synthesized from a variety of metallic and non-metallic materials (such as iron oxide, gold, and silica), consist of a variety of shapes (like rods, tubes, stars, spheres) under abundant construction paradigms (homogenous throughout, varying core-shell constructs, and various combinations and permutations of these concepts) and can be functionalized for use with various imaging modalities, such as PET, CT, MRI, ultrasound, and optical imaging [85]. Among the most popular and earliest-used nanoparticles for SLND purposes are superparamagnetic iron oxide particles. These particles intrinsically modify magnetic fields and are perfect candidates for use with MRI. They are especially useful in identifying lymph node metastases, as tumors take up the space of the macrophages that would otherwise occupy the nodes, thereby limiting the uptake of these particles in affected nodes [86]. In a seminal work, Harisinghani *et al*. intravenously injected 80 patients with lymphotropic superparamagnetic nanoparticles and observed neighboring nodes for metastases. The use of the nanoparticles allowed the researchers to identify metastases in all 33 patients which had them, and the technique had an overall sensitivity of 90.5% [87].

**Fig. 6:**
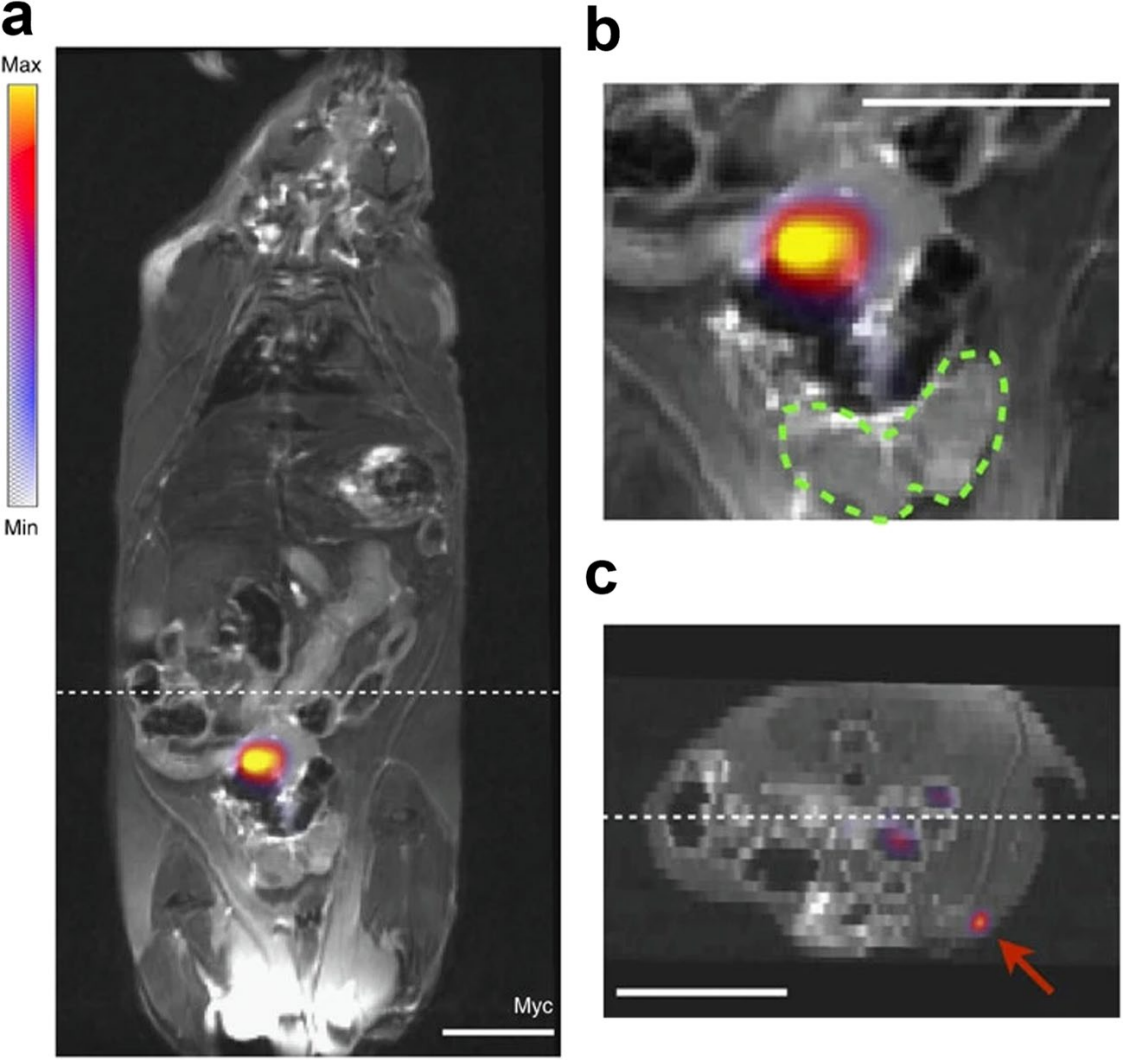
**a** Whole body coronal PET/MRI fused slice of [89] Zr-ferumoxytol in a draining lymph node, 6 h post-injection. **b** Magnified prostate region distinctly shows that the draining LN is outside of the prostate organ. The ventral prostate in this slice is delineated by a dashed green outline. **c** In the reconstructed axial orientation (located at the hashed marks on the coronal image), sensitive PET detection enables visualization of tracer in a more distant draining inguinal node (red arrow). In the clinical context, the detection of a draining LN outside of the pelvic bed would necessitate a widened field of biopsy sampling or therapeutic intervention. All scale bars are 0.8 cm in length. (Adapted with permission from Thorek et al., 2014, ©2014, Nature Communications, published by Springer Nature)

While this and similar papers have had an important impact in demonstrating the overall efficacy of nanoparticles as useful contrast agents, many of these initial studies did not highlight the multimodal potential of nanoparticles [88]. Subsequent studies, however, began exploring this realm further. For instance, a 2014 study by Thorek *et al*. utilized a radiolabeled iron oxide nanoparticle to identify draining lymph nodes in a mouse prostate cancer model using PET/MRI. Following administration of the nanoparticles directly into the mice’s prostates, researchers were able to observe drainage to the primary sentinel nodes as well as more distant structures, such as the inguinal nodes (Fig. 6) [89]. With the recent engineering advancements in multimodal imaging instrumentation, more and more hospitals around the U.S. have become equipped with state-of-the-art dual PET/MRI imaging capabilities. This multimodal approach now offers the combined advantages of each imaging technique and opens a whole new way to study the lymphatics.

**Fig. 7:**
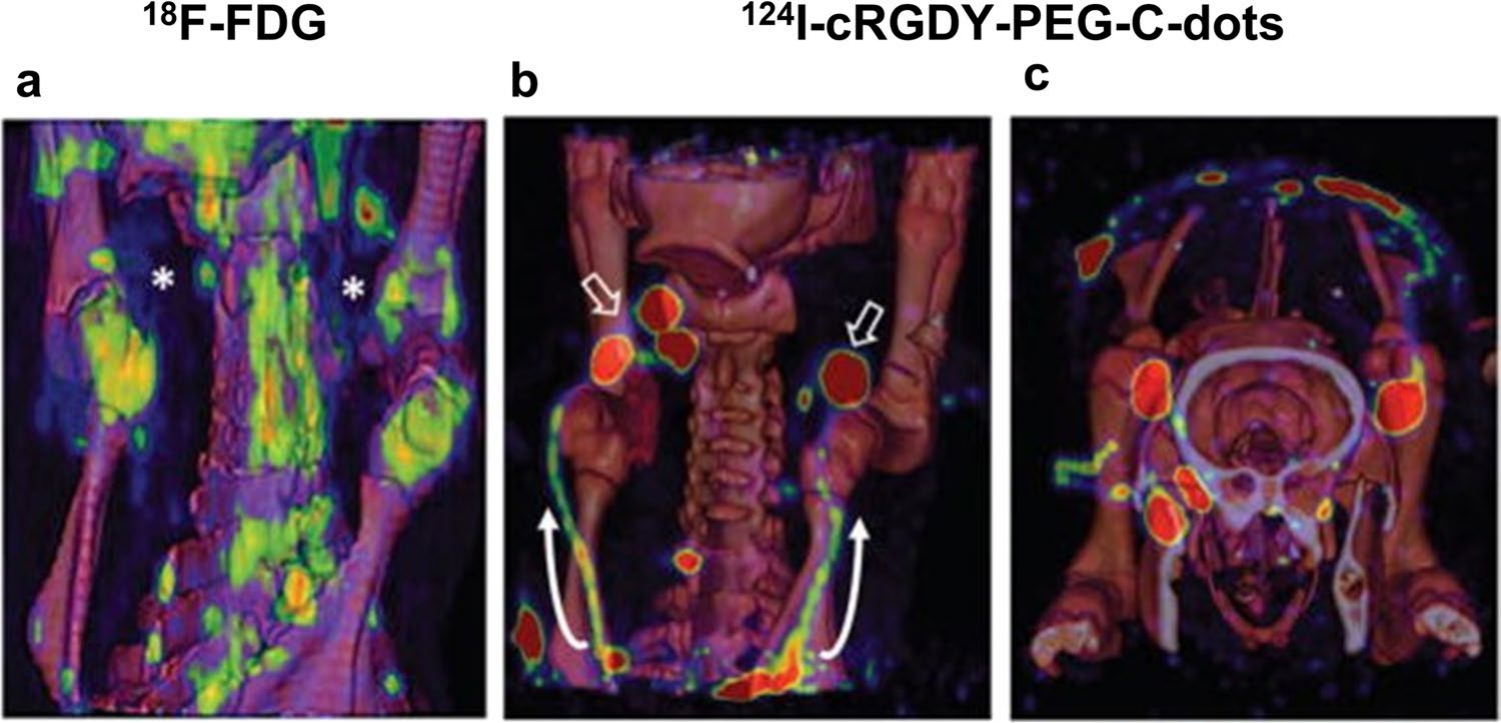
3D PET-CT fusion maximum intensity projection images integrated 18F-FDG and 124I-cRGDY-PEG-C dot PET-CT. **a** In the 18F-FDG PET-CT fusion image (coronal view) there is no evident nodal metastases (asterisks). Increased activity within bony structures is identified. **b**, **c** In contrast to the 18F-FDG image, the 124I-cRGDY-PEG-C-dot high-resolution PET-CT fusion images show coronal (**b**) and superior views (**c**) of bilateral metastatic nodes (open arrows) and lymphatic channels (curved arrows) within the neck following local injection of 124I-cRGDY-PEG-C dots. (Adapted with permission from Bradbury et al., 2013, ©2013, Integrative Biology, published by Oxford Academic)

Additional studies using differing nanoparticle types have also demonstrated the multimodal capabilities of nanoparticles. Among the most promising of these studies was a 2013 manuscript by Bradbury *et al*., in which the authors developed an optical-PET multimodal silica nanoparticle, termed ^124^I-cRGDY-PEG-C dots. These particles integrated Cy5.5 fluorophore into their cores while the shells/surfaces contained specific peptide-ligands to target tumors, PEG to reduce non-specific uptake of nanoparticles, and ^124^I to serve as the radiolabel. Using a melanoma mini-swine model, researchers were able to demonstrate improved detection of metastatic tumors in draining nodes by the C dots in comparison to sole use of ^18^F-FDG radiotracer (Fig 7) [90]. Following the publication of this study, the research group launched a first-of-its-kind clinical trial to assess the biodistribution, pharmacokinetics, and radiation dosimetry of the nanoparticles [91]. Following the conclusion of this safety study with favorable results, Bradbury’s group then conducted a non-randomized clinical trial on 24 melanoma patients from 2015 to 2018. During the trial, the authors found that there was a 0% false-negative rate in lymph node metastasis identification, a 90% concordance rate between Tc 99m sulfur colloid and the ^124^I-cRGDY-PEG-C dots in node-to-node identification, indicating great efficacy in SLND guidance. This, in combination with the practical advantages conferred by using the C dot construct, including improved depth resolution in comparison to small-molecule dyes and highly sensitive identification of SLN’s in otherwise low accessibility regions, highlight the immense promise that novel technologies such as nanoparticles have in the field moving forward [92].

### Lymphatic Involvement in Other Disease Conditions

While the lymphatics have been commonly implicated for their roles in lymphedema and cancer metastasis, recent literature utilizing various imaging techniques have begun to examine their role more thoroughly in a host of other disease states. Given their status as a drainage and trafficking system, particularly for immune cells and antigens, the lymphatics have been implicated in several autoimmune conditions, including rheumatoid arthritis (RA). RA’s impact on the lymphatics was identified quite early, with several case reports from the late 1960s and on indicating lymphedema as a rare complication of the condition [93]. A 2007 study by Proulx *et al*. investigated lymphangiogenesis in an arthritis mouse model using contrast-enhanced MRI, noting an increase in lymphatic vasculature at the draining lymph nodes and synovia of the mice knees; however, this increase persisted despite reduction in inflammation following anti-tumor necrosis factor therapy (anti-TNF) [94]. Similarly, a 2010 study by Zhou and colleagues in a mouse model found the same increase in lymphatic vasculature without a corresponding increase in lymphatic flow, implying a level of poor functionality [95]. The same group then used MRI monitoring of lymphatic flow to demonstrate that the use of AAV-VEGF-C (an adenovirus vector delivering a lymphatic growth factor) improved drainage from local inflammations, helping mitigate damage resulting from the arthritic phenotype [96]. Subsequent studies have gone on to explore other impacts of RA on the lymphatics as well as other associated therapies. For instance, the 2013 work of Li *et al.* was able to use MRI, NIRF, and immunofluorescent intravital imaging to demonstrate that B-cell depletion therapy (BCDT) effectively increased lymphatic flow and removed B-cells from lymphatic spaces in the draining lymph node to relieve symptoms of RA [97].

In addition to their link to the immune system, the lymphatics also play a key role in the absorption of dietary lipids. As such, it is not altogether surprising that lymphatic dysfunction has also been tied to obesity. One of the most straightforward links between the lymphatics and obesity is lymphedema. Work by Green *et al*. in 2012 and 2015 suggested that obesity (notably in scenarios where a patient’s BMI exceeds 50–60) could be a cause of lymphedema due to buildup of adipose tissue disrupting lymphatic flow [98, 99]. Additional work by the same group using lymphoscintigraphy to visualize flow demonstrated sustained impairment in a severely obese patient following a sleeve gastrectomy [100]. While these studies helped establish the presence of an association, the exact mechanism of obesity’s impact on lymphatic dysfunction was unclear. A 2013 study by Weitman *et al*. revealed that obesity in a mouse model resulted in decreased size of draining nodes, reduced extent of lymphatic vascularity, and impaired dendritic cell migration [101]. Similarly, work by Savetsky *et al.* found that uptake of radiotracer by lymph nodes was decreased in obese mice; however, they also found that obese mice with lymphedema had 2.5 times the CD4+ cell and three times the CD45+ cell inflammatory responses relative to the lean control with lymphedema [102]. In addition to obesity’s exacerbation of lymphatic dysfunction symptoms, lymphatic functionality has been shown to impact obesity. In a 2016 study using several different lymphatic imaging techniques including NIRF, Escobedo *et al*. attempted to restore lymphatic functionality in *Prox1* heterozygous mice. These mice exhibit an obese phenotype due to excessive leakage of chyle from their lymph vessels, resulting in a buildup of adipose tissue. Escobado and colleagues were able to demonstrate that restoration of lymphatic functionality helped rescue the adult-onset obesity in the mice [103]. Additionally, using intravital imaging, Zhang *et al*. were able to demonstrate that blocking chylomicron uptake by zippering lacteal junctions helped make mice resistant to obesity and re-establishing their permeability restored chylomicron uptake, suggesting that targeting these junctions could be a potential target of therapeutics aiming to mitigate obesity [104].

Due to this lipid-transport role, the lymphatics have also been implicated in the formation of atherosclerotic plaques and heart disease in general. Work by Lim *et al*. and Martel *et al*. with *Apoe*-deficient mice demonstrated that the lymphatics were essential in the reuptake of cholesterol from tissues to the blood circulation (a phenomenon known as reverse cholesterol transport, or RCT), indicating that restoration of any disrupted lymphatic function could help remove cholesterol from atherosclerotic plaques [105, 106]. In 2014, Vuorio *et al*. studied the effect of lymphatic insufficiencies on the development of atherosclerotic plaques by crossbreeding two different types of lymphatic deficiency mouse models with two different types of atherosclerotic models. They found that the lymphatic insufficient and atherosclerotic offspring had significantly higher plasma cholesterol concentrations relative to the control atherosclerotic mice and that their lesion development proceeded more rapidly. The researchers also found less lymphatic vasculature in the lesions of the crossbreeds in comparison to those of the control mice, suggesting that the lymphatic insufficiencies helped exacerbate the development of the plaques [107]. In addition to its role in the development of plaques, the lymphatics have also been directly tied to myocardial infarctions (MI). For instance, a 2018 study found that stimulation of lymphangiogenesis in a mouse model using VEGF-C following MI resulted in mitigation of the inflammatory response due to enhanced clearance and drainage, making targeting the lymphatics an interesting potential therapeutic avenue for cardiac tissue preservation [108].

Following the previously mentioned discovery of meningeal lymphatic vessels, there has also been significant interest in uncovering their potential role in a host of neurodegenerative diseases, including Alzheimer’s. In a 2018 study, Da Mesquita *et al*. found that ablating these meningeal lymphatics resulted in disruption of CSF and ISF transport via the paravascular (glymphatic) system and subsequently resulted in cognitive impairment as indicated by significant decrease in tracer drainage from the CSF to the deep cervical lymph nodes in the treatment group relative to controls. Researchers also found that this ablation in a transgenic Alzheimer’s model resulted in increased amyloid-β plaque deposition in the meninges, indicating that lymphatic disruption can exacerbate symptoms. This makes the meningeal lymphatics a potential area of focus for treatment developments to help potentially slow down the advance of Alzheimer’s [109, 110]. While studies exploring the role of the lymphatics in glymphatic efficacy and amyloid-β trafficking have been only directly performed in animal models, researchers in 2021 through a systematic review were able to identify amyloid-β in high degree of abundance in cervical lymph nodes relative to inguinal lymph nodes, allowing them to conclude that there was glymphatic involvement in amyloid-β clearance in humans [111].

In summary, the impact of the lymphatics extends into an enormous range of pathologies. Beyond the disorders discussed in more depth above, the lymphatics have also been implicated in inflammatory bowel disease, ocular disorders, strokes, type 2 diabetes, along with many other conditions [112, 113]. The use of lymphatic imaging has been crucial in the discovery of many of these links, and it is apparent that refinement of these techniques will help improve our identification of any new associations between the lymphatics and human pathologies, the search for novel treatments, and understanding of the mechanisms of any lymphatic-focused therapies.

## Conclusion

Despite many recent and ongoing discoveries concerning the lymphatic system, the field remains ripe for further exploration, with respect to fundamental understanding of lymphatic architecture and physiology as well as the role of the lymphatics in various pathologies. Imaging is a crucial tool in bolstering this progress, and so the development of novel contrast agents and imaging tools remains of the utmost importance. Using new platforms, such as nanoparticles with multimodal imaging capabilities, could allow researchers and clinicians to extract greater information while also streamlining experimental procedures. While these new technologies have already begun to demonstrate their effectiveness, additional work is necessary to validate their efficacy in comparison to more traditional methods and ensure thorough translation to clinical settings.
